# Co-delivery of amphotericin B and pentamidine loaded niosomal gel for the treatment of *Cutaneous leishmaniasis*

**DOI:** 10.1080/10717544.2023.2173335

**Published:** 2023-02-01

**Authors:** Adnan Anjum, Kanwal Shabbir, Fakhar Ud Din, Shumaila Shafique, Syed Saoud Zaidi, Ali H Almari, Taha Alqahtani, Aleena Maryiam, Muhammad Moneeb Khan, Adel Al Fatease, Sidra Bashir, Gul Majid Khan

**Affiliations:** a Nanomedicine Research Group, Department of Pharmacy, Faculty of Biological Sciences, Quaid-I-Azam University, Islamabad, Pakistan; b Department of Pharmacy, Faculty of Biological Sciences, Quaid-I-Azam University, Islamabad, Pakistan; c Dow College of Pharmacy, Faculty of Pharmaceutical Sciences, Dow University of Health Sciences Karachi; d Department of Pharmaceutics, College of Pharmacy, King Khalid University, Abha, Saudi Arabia; e Department of Pharmacology, College of Pharmacy, King Khalid University, Abha, Saudi Arabia; f Islamia College University, Peshawar, Pakistan

**Keywords:** *Cutaneous leishmaniasis*, niosomes, amphotericin B, pentamidine, AmB-PTM-NIO-Gel, topical delivery

## Abstract

Topical drug delivery is preferable route over systemic delivery in case of *Cutaneous leishmaniasis* (CL). Among the available agents, amphotericin B (AmB) and pentamidine (PTM) showed promising result against CL. However, monotherapy is associated with incidences of reoccurrence and resistance. Combination therapy is therefore recommended. Thin film hydration method was employed for amphotericin B-pentamidine loaded niosomes (AmB-PTM-NIO) preparation followed by their incorporation into chitosan gel. The optimization of AmB-PTM-NIO was done via Box Behnken Design method and in vitro and ex vivo analysis was performed. The optimized formulation indicated 226 nm particle size (PS) with spherical morphology, 0.173 polydispersity index (PDI), −36 mV zeta potential (ZP) and with entrapment efficiency (EE) of 91% (AmB) and 79% (PTM), respectively. The amphotericin B-pentamidine loaded niosomal gel (AmB-PTM-NIO-Gel) showed desirable characteristics including physicochemical properties, pH (5.1 ± 0.15), viscosity (31870 ± 25 cP), and gel spreadability (280 ± 26.46%). In vitro release of the AmB and PTM from AmB-PTM-NIO and AmB-PTM-NIO-Gel showed more prolonged release behavior as compared to their respective drug solution. Higher skin penetration, greater percentage inhibition and lower IC50 against the promastigotes shows that AmB-PTM-NIO has better antileishmanial activity. The obtained findings suggested that the developed AmB-PTM-NIO-Gel has excellent capability of permeation via skin layers, sustained release profile and augmented anti-leishmanial outcome of the incorporated drugs.

## Introduction

Leishmaniasis is a parasitic disease caused by parasites found in different species of Leishmania (Shirian et al., 2013; Dar et al., 2018). Despite being among the top 10 individual disease burden, Leishmaniasis has been ignored globally (Alvar et al., 2012). Almost 1.5 million new cases of *Cutaneous Leishmaniasis* (CL) are being reported annually (Dar et al., 2018). The main hindrance in controlling the leishmaniasis is non availability of vaccine, safe and effective pharmacological agents and special diagnostic equipment’s (Hailu et al., 2016). CL is a major health risk that can cause variety of diseases ranging from self-healing infections to chronic disfiguring disease (Scott & Novais 2016). CL is characterized by the formation of abscess and chronic inflammation of skin (Rabia et al., 2020). It varies from tinny nodules to plaques and ulcer like lesion on the surface of skin (Batool et al., 2021).

**Figure 1.**
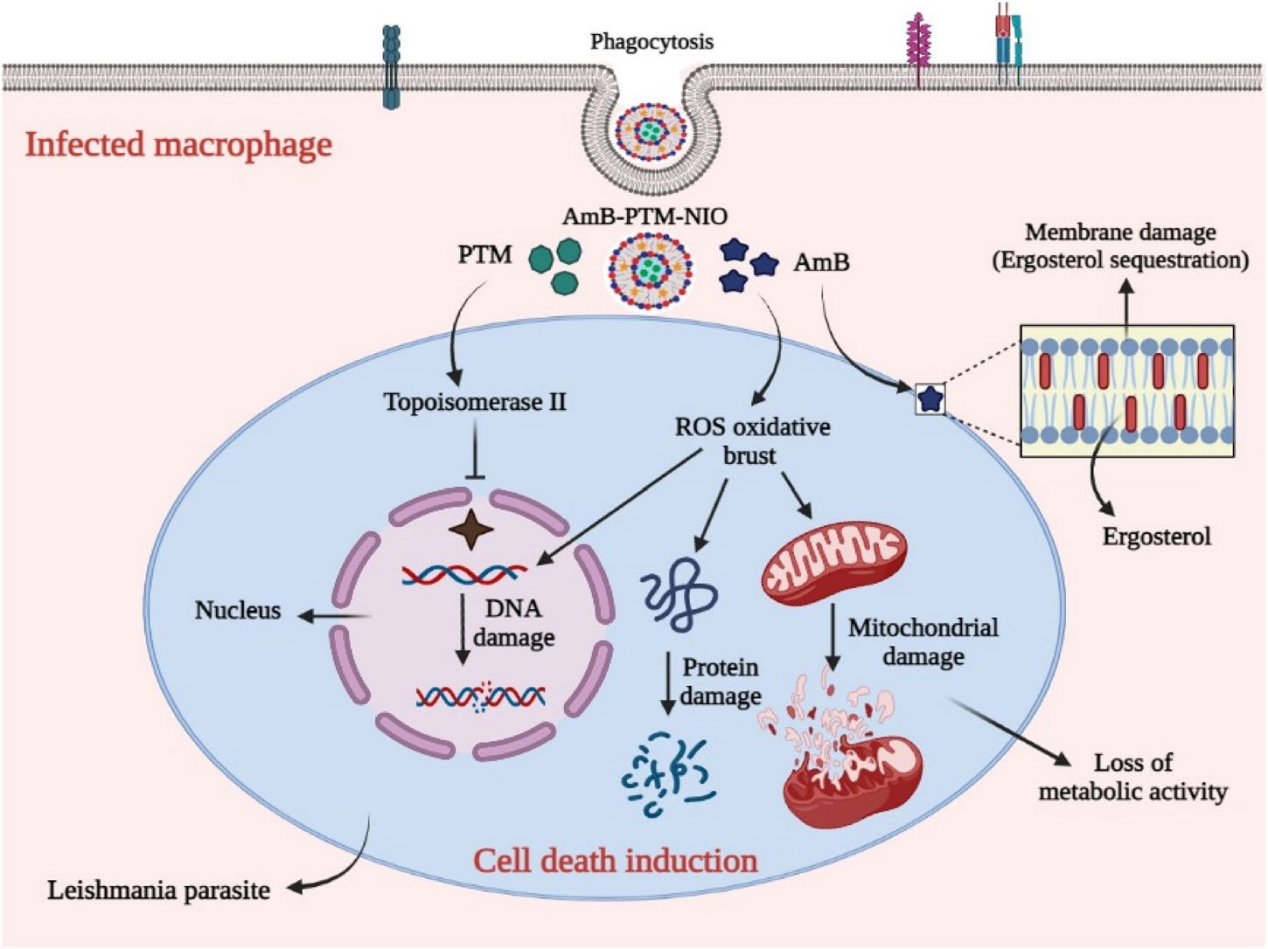
Schematic diagram of mechanism of Leishmania effected macrophage targeting via AmB and Pentamidine.

Amphotericin B (AmB) is a potential agent for the treatment of various endemic and opportunistic fungal infections and was launched into clinical practice in 1959 (Adler-Moore et al., 2019). Besides, AmB is also considered as most effective agent for the treatment of CL and numerous drug delivery systems have been studied in this regard (Lanza et al., 2019). AmB kills parasites by making pores in the cell membrane (Laniado-Laborín & Cabrales-Vargas 2009; Lanza et al., 2019; Mostafavi et al., 2019; Carolus et al., 2020). However, AmB dose-dependent renal toxicity and infusion associated reactions limited its clinical use (Groll et al., 2019). Chemically pentamidine (PTM) is an aromatic diamidine which have anti parasitic activity. After its synthesis in 1940, it was mainly used for the treatment of human African *trypanosomiasis*, infection caused by *Pneumocystis jirovecii*, and also for Visceral Leishmaniasis (VL) and CL (Gadelha et al., 2018). PTM has a key role in the treatment of all forms of leishmaniasis in a situation when first line agents fail, or when the patient is unable to tolerate the side effects of the first line agents. It is also a suitable candidate for combination therapy (Piccica et al., 2021). PTM interferes with the synthesis of polyamine, RNA polymerase activity and inhibits the protein, nucleic acid, phospholipid and folate synthesis. It also behaves as anti-inflammatory and xenobiotic agent and additionally blocks the NMDA receptor (Hafiz & Kyriakopoulos, 2020). Intra-lesion (IL) injection of PTM have less local side effects than IL injection of the antimonial (Soto et al., 2016). An illustration of the mechanisms of both the antileishmanial agents (AmB and PTM) is presented in Figure 1.

Niosomes are comprised of nonionic surfactants and cholesterol which are cost effective and at the same time safe for use in biomedicine (Wagh & Deshmukh, 2010; Bartelds et al., 2018). When applied topically, niosomes increases the drug residence time at the stratum corneum (SC) and epidermal layer and minimizes the systemic availability of drug. They are compatible and non-immunogenic to the biological system. Niosomes are good candidates for targeted, controlled release and sustained release drug delivery systems and at the same time can be administered by various routes e.g. ocular, transdermal, topical and parenteral etc. They also give better drug entrapment efficiency of both hydrophilic and hydrophobic drugs (Bhardwaj et al., 2020). Conventional dosage forms such as ointments, creams and gels were used for the topical treatment in most of the studies which showed promising results, but with variable efficacy and sometimes disappointing output was seen, because skin penetration was the rate limiting step. In order to cope with this problem nano carriers showed better results (Carneiro et al., 2012).

Due to side effects reported with oral and parenteral route of administration, WHO recommended the topical route of administration for the treatment of CL (Organization, 2010). Although, topical route avoids adverse effects associated with systemic absorption, yet creates hindrance in penetration of drug through the stratum corneum (SC) and this problem is best resolved by nanoparticles (Jamshaid et al., 2021). Similarly, Salim *et al,* reported the promising result of chitosan based rifampicin and vancomycin transferosomal gel against CL via topical route of administration (Salim et al., 2020). Bezerra-Souza *et al.,* demonstrated that topical treatment of animals with butenafine showed good results with decrease in lesion size and parasite load. At the same time butenefine gel presented similar results as the efficacy shown by glucantime administered by intra leisional route (more side effects and drug resistance problem) (Bezerra-Souza et al., 2021). Herein, AmB-PTM-NIO were incorporated into chitosan gel to improve the withholding time of nanocarriers at the site of infection and enable AmB-PTM-NIO to penetrate skin layers for long time period. AmB-PTM-NIO were engulfed by macrophages after crossing skin layers, where they promoted healing by killing the leishmanial strains. Thus, we report AmB-PTM-NIO loaded gel with targeted drug delivery inside macrophages with better anti leishmanial activity.

## Materials and methods

### Chemicals and reagents

AmB and PTM were purchased from Macklin Biochemical Co., Ltd. (Shanghai, China). Cholesterol, span 60, span 80, methanol, ethanol, potassium chloride and sodium chloride were purchased from Sigma-Aldrich (Heidenheim, Germany). Chloroform, potassium dihydrogen phosphate and disodium hydrogen phosphate were purchased from Duksan Pure Chemicals (Ansan-si, South Korea). Roswell park memorial institute (RPMI-1640) medium, was obtained from Thermo Fisher Scientific, USA. Medium199, fetal bovine serum, penicillin and streptomycin were purchased from m BDH laboratory, England. Rest of the chemicals used were purchased from local manufacturers.

### Animals and parasites

Male albino rats weighing of 100-120 g were purchased from National Institute of Health (NIH), Islamabad. According to the NIH’s recommendations for the management and care of laboratory animals, the animals were housed in conventional facilities. Moreover, ARRIVE guidelines were followed for animal study. Normal rat food and tap water was provided to the animals. Bioethical approval was obtained from bioethical committee of Quaid-i-Azam University, Islamabad, Pakistan (Protocol no BEC-FBS-QAU2021-320). *L. tropica* were procured from Khyber Medical University Peshawar KPK, Pakistan.

### AmB-PTM-NIO preparation

AmB-PTM-NIO were prepared by film hydration method described by Seguella et al. (2020) with minor modifications. In a round-bottom flask, span 60 and span 80 with different ratios were dissolved in a mixture of ethanol and chloroform (2:3) along with AmB (previously dissolved in acidified methanol). Organic solvents were evaporated using rotary evaporator by maintaining temperature at 60 °C for time period of 15 minutes. It produced a thin film on the interior walls of the flask. Hydration of the film was done with 10 mL of phosphate buffer (pH 7.4) containing PTM at 60 °C for 1 hr resulting in the formation of AmB-PTM-NIO. Controlled niosomes were also prepared using same procedure as shown in (Supplementary Figure 1) (Mostafavi et al., 2019; Seguella et al., 2020).

### AmB-PTM-NIO-gel preparation

2% (w/v) chitosan gel was prepared and used for the loading of AmB-PTM-NIO. Blank chitosan gel was prepared by dissolving the medium weight chitosan in 1% (v/v) acetic acid solution with continuous stirring. For preparation of AmB-PTM-NIO-Gel, 3 mL of 1% acetic acid was added into 100 mg of the chitosan under constant magnetic stirring. Then, 2 mL of phosphate buffer saline PBS (pH 7.4), which contained pellets of AmB-PTM-NIO was added to the mixture to make the final volume equal to (5 mL). Finally, a homogenous gel was prepared by continuous stirring of the mixture for 2 hr (Sohrabi et al., 2016).

## Characterization

### Optimization of blank niosomes by Box-Behnken design

Initially screening trials were carried out in order to find the effect of different parameters on the final properties of niosomes. It was observed that span 60, span 80 and cholesterol had a potential effect on the desired characteristics of the niosomes which in turn can interfere with the dermal delivery of niosomes through the skin. On the basis of these observational studies the concentration of each factor was set accordingly. For the optimization of blank niosomes, Box-Behnken Design Expert® (version 12, Stat-Ease Inc., USA) software was used. The software generated 17 experimental runs. Cholesterol, span 60 and span 80 were independent variables while particle size, PDI and zeta potential were dependent variables (Imran et al., 2022).

### AmB-PTM-NIO particle size, PDI and zeta potential determination

Niosomes were characterized in terms of PS, PDI and ZP using Zeta sizer ZS90 (Malvern instruments, Worcestershire, UK). The sample was prepared by taking 10 microliter of formulation and diluted with distilled water up to 1 ml. Sample was run in the zeta sizer for size determination and triplicate readings were obtained. ZP was determined by inserting Dip cell into the sample. Results were noted in triplicate for each sample (ud Din et al., 2019; Batool et al., 2021).

### Determination of encapsulation efficacy (EE)

Indirect method was employed to calculate %EE of prepared niosomes. Briefly, niosomal formulation containing AmB and PTM was subjected to centrifugation for 1 hr at 15000 rpm. The supernatant was removed carefully. A spectrophotometer (HALO DB-20 UV-VIS double beam) was used to observe the absorbances at 406 and 268 nm, for AmB and PTM respectively. %EE was calculated using following formula (Khan et al., 2021):

(1) $$\%EE=\;\frac{\mathrm{Total}\;\mathrm{amount}\;of\;\mathrm{drug}-\mathrm{unentrapped}\;\mathrm{drug}}{\mathrm{Total}\;\mathrm{amount}\;of\;\mathrm{drug}\;}\;\times 100\;$$

### Transmission electron microscopy (TEM)

AmB-PTM-NIO morphology, homogeneity and size was further confirmed by TEM (TEM, Hitachi H- 7600; Tokyo, Japan) analysis at 100 kV accelerating voltage. Few drops of optimized niosomal dispersion were taken that was 50 times diluted previously with double distilled water onto a 400- mesh carbon film coated copper grid. The sample was negatively stained with 1% phosphotungestic acid before drying of carbon film on grid for 10 sec. Sample was air dried before TEM analysis (Khan et al., 2022; Zahid et al., 2022).

### Characterization of AmB-PTM-NIO-Gel

#### Organoleptic evaluation and pH determination

Color, appearance and homogeneity of AmB-PTM-NIO-Gel were evaluated visually. pH of AmB-PTM-NIO-Gel was determined with digital pH meter (PH 700 EUTECH instruments, Germany) by dissolving 1 g of gel in 50 ml double distilled water followed by checking pH of the gel using (Dar et al., 2018).

#### Rheology

Viscosity of the gel was measured by using Brookfield cone and plate rheometer (Brookfield Engineering Laboratories Inc., Middleborough, MA) at room temperature using spindle CPA 52 Z. A sufficient amount of gel was added to the receiving chamber and runs were carried out there at temperature of 25 °C with shear rates ranging from 1 to 100 s ^− 1^. A rheogram was plotted between shear rate and viscosity of AmB-PTM-NIO-Gel (Din et al., 2021; Jiao et al., 2023).

#### Drug content

To determine the drug content of the AmB-PTM-NIO-Gel, 1 g of gel was dissolved into 100 ml of PBS and kept for 24 hr. After 24 hr, AmB-PTM-NIO-Gel and PBS mixture was stirred for 30 min by using magnetic stirrer (Eisco Scientific, North America). Solution was then filtered, and absorbance was observed with UV-visible spectrophotometer (Batool et al., 2021).

#### Gel spreadability

The gel spreadability was measured by making a circle of 1 cm diameter on a glass slide and 0.5 g of gel was placed inside the drawn circle. A second glass slide was placed over it and a weight of 0.5 kg was placed on second slide. After that new diameter was drawn and the spreadability of the AmB-PTM-NIO-Gel and blank gel was calculated by using the equation given below (Salim et al., 2020).

(2) $$S=d^{2}\times \frac{\pi }{4}$$

Where, ‘S’ is representing the spreadability of gel, ‘d’ is representing the diameter after placing the weight (mm).

### In vitro drug release study

Dialysis bag diffusion method was employed to perform in vitro release study of the PTM-NIO-Gel and compare with PTM-NIO, AmB-NIO and their respective solutions (Khan et al., 2021). All the comparative formulations had equivalent amount of PTM and AmB equal to 10 mg and 20 mg, respectively. This amount was selected based on the optimization process as discussed in Table 2. Shaking water bath apparatus (Memmert SV 1422) was used for in vitro drug release studies. Drug release study was carried out at pH 7.4 and 5.5 to imitate the physiological pH of blood and macrophages respectively. Temperature of the system was maintained at 37 °C. Approximately 50 ml of PBS were added in 100 ml beakers and placed in the water bath for temperature equilibration. AmB-PTM-NIO formulation, AmB-PTM-NIO-Gel, AmB suspension and PTM solution were added into dialysis bags and bags were suspended into beakers containing release media. From each beaker, 2 ml of sample was collected after time interval of 0.25, 0.5, 1, 2, 4, 6, 12 and 24 hr and replaced with 2 ml of fresh PBS. UV-spectrophotometer (HALO DB-20 UV-VIS double beam) was used to evaluate samples at respective wavelengths and graph was plotted between % cumulative release of drug and time (Dar et al., 2018).

**Table 2.** Optimization of drug loaded niosomes.

F. code	Chl. (mg)	Span-60 (mg)	Span-80 (mg)	AmB (mg)	PTM (mg)	PS (nm)	PDI	ZP (mV)	AmB (%EE)	PTM (%EE)	Physical stability
D1	20	25	25	3	3	238.2 ± 2.38	0.182 ± 0.045	−42 ± 2.38	91 ± 1.42	59 ± 3.14	Clear
D2	20	25	25	2	3	220.3 ± 3.11	0.197 ± 0.032	−40.1 ± 3.15	93 ± 2.51	61 ± 3.65	Clear
D3	20	25	25	2	4	226.4 ± 2.14	0.173 ± 0.021	−36.6 ± 2.31	91 ± 2.87	79 ± 2.24	Clear
D4	20	25	25	2	5	292.8 ± 2.56	0.201 ± 0.025	−33.2 ± 3.87	90 ± 3.51	74 ± 3.16	Clear
D5	25	25	25	2	6	284.7 ± 2.87	0.184 ± 0.039	−31 ± 2.44	90 ± 3.56	75 ± 2.21	Clear

All the values here represent ± standard deviation; F.Code: Formulation code; Chl: Cholesterol; PS: Particle Size; PDI: Poly Dispersity Index; ZP: Zeta Potential; nm: Nanometer; mV: Millivolt; mg: Milligram EE: Entrapment Efficiency.

### Ex vivo permeation study

#### Skin preparation

Permeability of the AmB-PTM-NIO-Gel was determined and compared with AmB-PTM-NIO, AmB dispersion and PTM solution using rats’ skin. Momentarily, twelve rats were euthanized followed by shaving their abdominal regions before permeation analysis. They were classified into four groups; each carrying 3 rats and were used for the permeation study of respective groups. Their skin was eliminated trailed by the removal of subcutaneous layers, fats and washing with PBS (pH 7.4). Finally, the washed skin was enfolded in aluminum foil, and stored at −80 °C. At desired time, skin was taken out, defrosted at room temperature by drenching in PBS (pH 7.4) for 2 hrs (Zahid et al., 2022).

#### Skin permeation study

Ex vivo permeation study was carried out by Franz diffusion cell apparatus (Model: T9-CB-71026, Make: PermeGear, Inc. USA) having 0.77 cm^2^ permeation area and a 5.2 ml receiving compartment. Temperature of apparatus was maintained at 32 °C and freshly excised rat skin was clipped between the donor and receiver compartment. In receiver compartment PBS was filled and 300 rpm stirring rate was maintained. The AmB dispersion, PTM solution, AmB-PTM-NIO and AmB-PTM-NIO-Gel were kept in the donor section. The permeation analysis was executed for a period of 24 hrs. The sample was drawn at 0.25, 0.5, 1, 2, 4, 6, 12 and 24 hrs and replaced with equal amount of fresh PBS. Collected sample was analyzed by UV-spectrophotometer (Dar et al., 2018). Graph with % drug permeation (y-axis) and time of sampling (x-axis) were plotted by using GraphPad prism® v.9.0. The steady state flux (Jss) (µg/cm^2^/h), enhancement ratio (ER) and permeation coefficient (Kp) (cm^2^/h) were determined using the given formulas (Rabia et al., 2020):

(3) $$Jss=\frac{\mathrm{Amount}\;of\;\mathrm{permeated}\;\mathrm{drug}\;}{\mathrm{Time}}$$

(4) $$ER=\frac{\mathrm{Jss}\;of\;\mathrm{the}\;\mathrm{formulation}}{\mathrm{Jss}\;of\;\mathrm{the}\;\mathrm{pure}\;\mathrm{drug}\;\mathrm{dispersion}}$$

(5) $$Kp=\frac{\mathrm{Jss}}{\mathrm{initial}\;\mathrm{amount}\;of\;\mathrm{NTZ}\;in\;\mathrm{donor}\;\mathrm{cell}}$$

### Skin structure evaluation

In order to find any potential change or damage in the skin tissues, FTIR analysis was accomplished. Usually, the skin has intact lipid bilayer structure in various layers, however, disruption of these layers may occur when stress is applied which may results in changing the structure of lipids specifically in the epidermis layer. In this study, epidermis of the skin was isolated using manual method. Briefly, skin was treated with isopropyl alcohol and dipped in pre-heated water (60 °C). The epidermis was clipped between the donor and receiver compartment of Franz diffusion cell. AmB-PTM-NIO-Gel was placed on the epidermis for 4 hrs as reported earlier in permeation study. Then, the epidermis was isolated and placed in PBS in order to remove the excess formulation. After that FTIR analysis was performed at a wave number 4000–650 cm-1 for the identification of functional group of skin lipid (Rabia et al., 2020).

### Skin irritation and histopathological analysis

In vivo skin irritation study was performed to check the risk of any type of inducible skin irritation of AmB-PTM-NIO-Gel by comparing it with positive and negative control. The Draize scoring system was used to evaluate skin after treatment. The rats were divided into three groups (positive, negative and gel treated group). Formalin (0.8%) treated group was considered as positive control while untreated rats were kept in negative control group. Rats skin was observed for any sign of redness or edema and scoring was done at different time intervals (1, 24, 48, 72 hr). For further verification of results histopathological examination was also done. Cryostat microtome slices of skin samples were followed by microscopic inspection (Rabia et al., 2020; Batool et al., 2021).

### Antileishmanial Assay in in vitro setting

Antileishmanial assay was performed in in vitro setting using diphenyl-terazolium (MTT) colorimeter. Momentarily, M-199 medium containing fetal bovine serum (1% FBS) was prepared and added with penicillin (100 IU/mL) and streptomycin sulfates (100 µg/mL). Then the parasites (1 × 10^6^ promastigotes/mL) seeded in each well of 96-well plate comprising 20 µL of sample, at the concentration of 100 µg/mL (having <1% DMSO in PBS) were incubated at room temperature for 2h hrs. Neubauer hemocytometer was used for the counting of seeded promastigote. AmB and 1% DMSO was considered as positive and negative control respectively. The culture plates were incubated at temperature of 24 °C for 72 hrs. MTT solution (4 mg/ml) was then prepared and added into each culture plate. The plates were incubated again at 24 °C for 24 hrs. Supernatant of the plates were removed carefully after 4 hrs without disturbing sediment containing colored formazan. Dissolution of formazan crystals was achieved by adding DMSO (100 µL) to residue. Finally, microplate reader was used to measure the absorbance at wavelength of 540 nm. GraphPad prism® software was used for the calculation of IC50 (Gurjar et al., 2012; Mesquita et al., 2013). Below given formula was used to determine the growth inhibition rate:

(6) $$\mathrm{Growth}\;\mathrm{Inhibition}\;(\%)=\frac{\mathrm{Optical}\;\mathrm{density}\;of\;\mathrm{control}-\mathrm{Optical}\;d\mathrm{ensity}\;of\;\mathrm{test}}{\mathrm{Optical}\;\mathrm{density}\;of\;\mathrm{control}}\;\times 100\;$$

### Stability studies

The stability study was carried out for the optimized AmB-PTM-NIO over a period of 3 months by storing it at temperature 4 ± 2 °C and 30 ± 2 °C. The study was performed as per the international conference of harmonization (ICH) guidelines (Guideline, 2003). The freshly prepared AmB-PTM-NIO were divided into 6 samples (10 ml each) and were placed at their respective temperature conditions. At each specific time intervals (after 1 month), one sample was taken from each temperature storage conditions and was analyzed for PS, PDI, ZP and physical stability to find out any changes in their properties during the respective storage time period (Khan et al., 2021).

### Ex vivo cell uptake study

#### Harvesting of peritoneal macrophages

cRPMI (Cold Rosewell Park Memorial Institute) media was injected into the peritoneal cavity of the rats and abdomen was massaged for 10 seconds for the collection of resident macrophages. Media having resident macrophages was collected slowly and placed on ice. Finally, hemocytometer was used for cells counting that were placed and incubated for a period of 24 hrs with 5% CO_2_ supply at 37 °C at a concentration of 2 × 10^4^ million cells/well (Davies & Gordon, 2009).

#### Cell uptake study

Cell uptake study was performed on previously harvested macrophages in which macrophages were washed with PBS to remove the non-adherent cells. The AmB dispersion, PTM solution along with their niosomal preparation and AmB-PTM-NIO were added at a concentration of 50 µg/ml in 96-well plate and incubated for another 24 hrs in an environment with supply of 5% CO_2_ and at temperature of 37 °C was maintained. Free drug was removed by washing the macrophages with sterile PBS after separation of the supernatant. The macrophages were then scratched from the plate and cellular suspension was centrifuged at 4 °C with a speed of 3000 rpm for 5 minutes to get pellets of macrophages. Obtained pellets were treated with methanol and sonicated for 5 minutes. Finally, AmB and PTM content taken up the macrophages was calculated by spectroscopic examination (Chaubey & Mishra, 2014).

### Statistical analysis

Utilizing Design Expert® Software (version 12), statistical analysis and formulation optimization were carried out. However, for the examination of all other results, GraphPad Prism® (version 5) and Microsoft Excel 365 (version 2010) were utilized. Additionally one-way AVOVA and student t-test were also used. Studies with a p-value of 0.05 or less were statistically significant. The outcomes were also all presented as Mean ± Standard deviation.

## Results

### Optimization of niosomes by Box-Behnken design model

Response surface methodology (RSM) is a useful technique for the development and optimization of drug delivery system. This process entails mapping the response over the experimental domain and creating polynomial mathematical relations from a range of experimental designs. Different types of RSM deigns including D-optimal, Box-Behnken and central composite are used for statistical optimization of the formulations. Less experimental runs and time are required for this design that’s why it is considered as cost-effective technique compared to other techniques used for optimization of formulations (Abdelbary and AbouGhaly, 2015).

**Figure 2.**
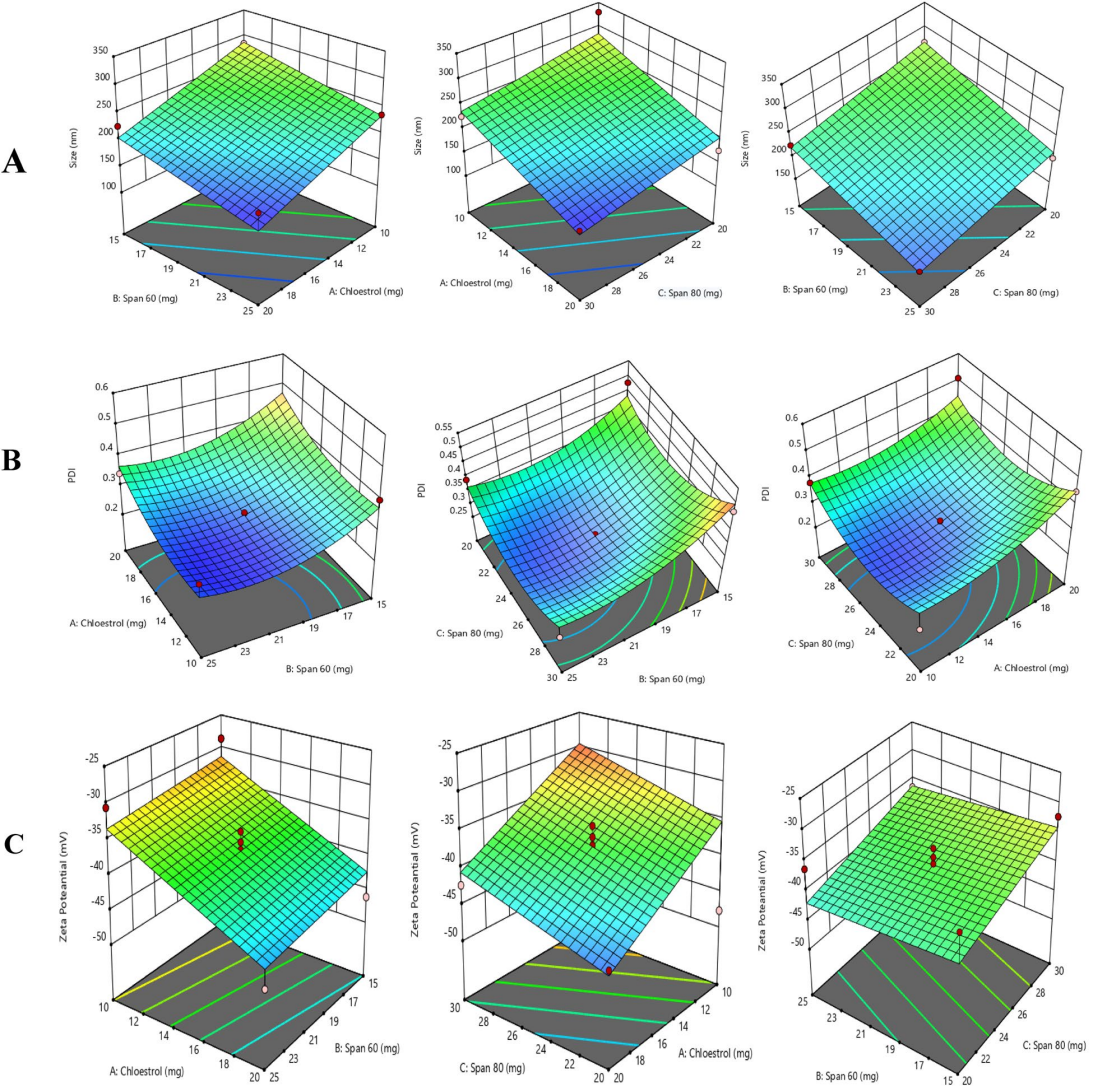
Optimization chart of the constituents of AmB-PTM-NIO via Box-Behnken Design. Effect of different variables on **(A)** Particle Size (PS); **(B)** Polydispersity index (PDI); **(C)** Zeta potential (ZP).

**Figure 3.**
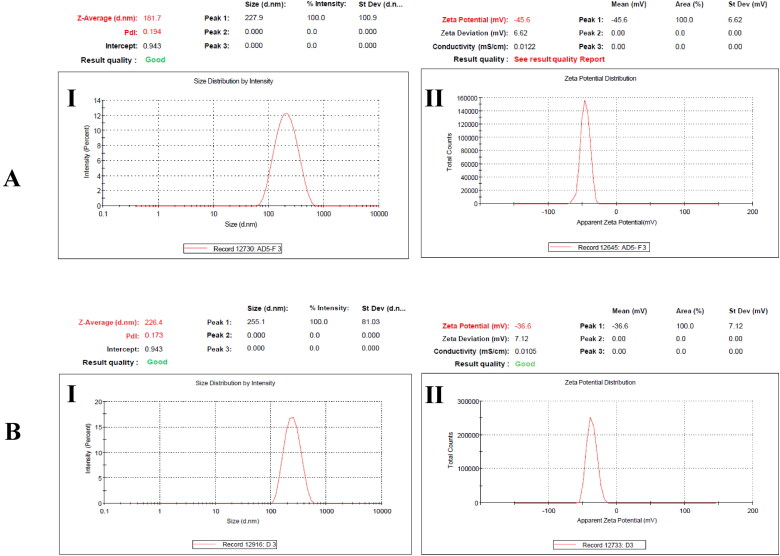
Evaluation of the Particle size, PDI and zeta potential **(A)** blank formulation **(B)** AmB-PTM-NIO; via DLS analysis. Data was taken in triplicate.

For the optimization of blank niosomes Box-Behnken Design expert® software (version 12, Stat-Ease Inc., USA) was used. Software generated 17 experimental runs. All the experimental runs generated by Box Behnken were performed and characterized in terms of PS, PDI and ZP and the results are shown in Table 1. The effect of independent variables on PS, PDI and ZP are shown by three-dimensional response surface graphs in (Figure 2). The optimized formulation was AD5 having particle size 181.3 ± 1.56 nm, PDI 0.195 ± 0.02 and zeta potential of −45.6 ± 1.93 mV as shown in (Figure 3).

**Table 1.** Formulation designed by Box-Behnken Design and observed responses for optimization of blank niosomes.

Formulation code	Independent variables				Dependent variables	
	Cholesterol (mg)	Span-60 (mg)	Span-80 (mg)	PS (nm)	PDI	ZP (mV)
AD1	10	20	20	330.0 ± 2.16	0.252 ± 0.06	−47.7 ± 1.28
AD2	15	20	25	201.5 ± 2.56	0.268 ± 0.02	−39.1 ± 1.68
AD3	15	20	25	206.9 ± 3.19	0.283 ± 0.04	−38.4 ± 2.24
AD4	20	15	25	225.1 ± 2.85	0.432 ± 0.09	−46.2 ± 1.57
AD5	20	25	25	181.3 ± 1.56	0.195 ± 0.02	−45.6 ± 1.93
AD6	15	25	20	204.1 ± 2.20	0.399 ± 0.04	−36.3 ± 1.58
AD7	15	15	30	225.2 ± 3.54	0.473 ± 0.07	−32.2 ± 2.59
AD8	15	20	25	209.1 ± 2.47	0.269 ± 0.06	−37.8 ± 2.33
AD9	10	25	25	235.0 ± 2.61	0.311 ± 0.02	−30.6 ± 2.99
AD10	20	20	30	180.3 ± 1.26	0.511 ± 0.09	−42.4 ± 2.67
AD11	15	25	30	170.7 ± 1.19	0.411 ± 0.07	−36.4 ± 3.52
AD12	20	20	20	175.0 ± 2.58	0.454 ± 0.03	−45.8 ± 2.32
AD13	15	15	20	270.4 ± 3.69	0.482 ± 0.05	−34.9 ± 1.33
AD14	10	20	30	225.1 ± 3.22	0.381 ± 0.02	−30.4 ± 2.81
AD15	15	20	25	207.8 ± 1.23	0.282 ± 0.06	−36.7 ± 2.66
AD16	10	15	25	285.4 ± 1.28	0.401 ± 0.03	−28.6 ± 1.22
AD17	15	20	25	205.1 ± 2.33	0.272 ± 0.04	−35.2 ± 2.33

All the values in the independent variables represent mean ± standard deviation; PS: particle size; PDI: poly dispersity index; ZP: zeta potential; nm: Nanometer; mV: Millivolt; mg: Milligram.

### Effect of independent variables on PS

PS considered as pivotal factor in case of topical formulation because it effects the skin permeation. The effect of independent variables was checked on PS by using Design expert® software having built in ANOVA. The p value <0.05 expressed a significant effect. Effect of independent variables i.e. cholesterol, span® 60 and span® 80 on PS is shown in (Figure 2A). The p value 0.0001 in case of cholesterol indicates its significant negative effect on PS i.e. increase in cholesterol concentration demonstrated decrease in PS, meaningfully. Similarly, span^®^ 60 and span^®^ 80 also have significant negative effects on PS as depicted by the p value which was observed (*p* < 0.05) in both cases.

### Effect of independent variables on PDI

PDI is another important parameter to be considered for the evaluation of homogeneity of formulation. PDI is a suitable indicator of the stability of a system. A nanosystem with low PDI have superior stability and PDI with higher value indicate instable system with heterogeneous formulation (Clayton et al., 2016). Our optimized formulation with PDI value of 0.195 ± 0.02 was in the optimal range. 3D graphs shown in (Figure 2B) indicates the effect of independent variables on the PDI. It was observed that, cholesterol concentration has a significant effect (*p* < 0.0359) on the PDI. The PDI was meaningfully increased as the cholesterol content was increased. Similarly, span^®^ 60 and span^®^ 80 also have significant effect on the PDI as depicted by the p value <0.05. Although, their concentration effect increased negatively by reducing the PDI values.

### Effect of independent variables on ZP

ZP also plays a key role in the stability of nanoparticles and identifying the charge on particles. Nanoparticles with high ZP leads to a stable system, while low ZP value results in aggregation of particles (Sameni et al., 2018). It is used as an indicator for stability of nanosystem, as the ZP increases, so does the stability (Cerqueira et al., 2017). Cholesterol has significant effect on ZP as indicated by p value <0.0041, while span^®^ 60 and span^®^ 80 didn’t show significant effect on ZP (*p > 0.05*) as shown in (Figure 2C)

### Optimization of drug loaded niosomes

After the optimization of blank niosomes, drug loaded niosomes were optimized in a series of experiments by adding various concentration of AmB and PTM. In this process, AmB and PTM were loaded into the niosomes at different concentration and resulting formulations were characterized in terms of PS, PDI and ZP, percentage entrapment efficiency (%EE) and physical stability (Table 2). As can be seen, the concentration of cholesterol, span^®^ 80 and span^®^ 60 were kept constant, whereas the concentration of both the drugs were varied. In first step, the PTM concentration was kept constant, whereas the concentration of AmB was varied (Table 2, D1–D2). The maximum %EE of the AmB was found as 91% and 93%, whereas the %EE of PTM was noted as 59% and 61%, demonstrating a non-significant effect of the AmB concentration on the final product. Moreover, reduction in PS was observed when the concentration of AmB was varied, however, no significant effect was detected on PDI and ZP.

In second step, the concentration of AmB was kept constant and that of PTM varied (Table 2, D2–D5). Results showed, a significantly increased %EE when the PTM content was changed from 3-4 mg, however upon further increase in the drug content, the %EE decreases. However, AmB %EE was not much effected. Similarly, an increase in PS was noted upon increase in the concentration of PTM. Moreover, it demonstrated a significant reduction in ZP, yet no change in the PDI was observed. As demonstrated in Figure 3(A), the PS, PDI and ZP of the blank formulation were respectively noted as 181.7 nm, 0.194 and −45.6 mV. However, upon loading of the drugs, the PS, PDI and ZP of the optimized formulation were found as 226.4 nm, 0.173 and −36.6 mV respectively (D3-Table 2 and Figure 3B).

**Table 5.** Stability study of optimized formulation at accelerated temperatures.

Time period	At temperature 4 °C ± 2 °C			At temperature 30 °C ± 2 °C				
	PS (nm)	PDI	ZP (mV)	Physical stability	PS (nm)	PDI	ZP (mV)	Physical Stability
0 Day	228.12 ± 1.39	0.195 ± 0.05	−36.32 ± 1.94	Stable	228.12 ± 1.39	0.195 ± 0.08	−36.32 ± 1.94	Stable
1 Month	228.1 ± 2.58	0.196 ± 0.03	−36.29 ± 1.56	Stable	231.10 ± 3.11	0.243 ± 0.04	−35.11 ± 1.31	Stable
2 Month	230.5 ± 2.04	0.203 ± 0.07	−35.54 ± 1.52	Stable	235.21 ± 4.39	0.294 ± 0.09	−32.87 ± 2.61	Stable
3 Month	235.2 ± 1.14	0.232 ± 0.02	−34.28 ± 1.37	Stable	239.34 ± 5.68	0.258 ± 0.05	−32.59 ± 3.22	Stable

Here, PS: particle size; PDI: polydispersity index; ZP: zeta potential; ±: standard deviation *n* = 3.

### Transmission electron microscopy (TEM)

**Figure 4.**
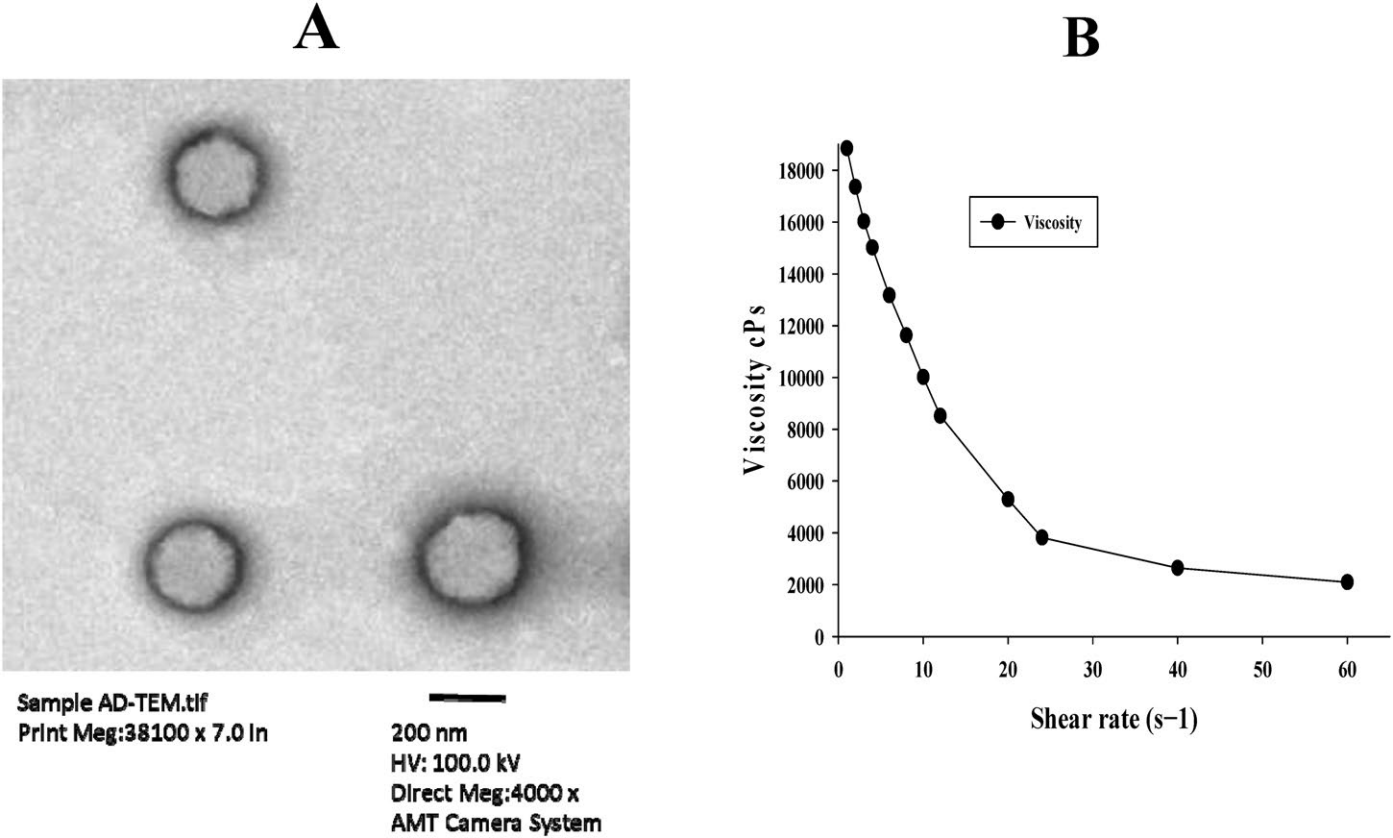
(A), Transmission electron microscopy (TEM) image of AmB-PTM-NIO (6000X). (B) Evaluation of the effect of AmB-PTM-NIO shear rate on viscosity.

TEM analysis of the optimized AmB-PTM-NIO formulation is represented in (Figure 4A). It showed well segregated and well identified particles with spherical shape and smooth outer layer. Moreover, the particles were well distributed in the nano system. In addition, TEM analysis further endorsed the particle analysis reports obtained from the Zeta sizer.

### Physicochemical and rheological study of AmB-PTM-NIO-Gel

Physicochemical characterizations of AmB-PTM-NIO-Gel and blank gel were carried out and it was observed that appearance of AmB-PTM-NIO-Gel was clear, opaque with uniform homogeneity having pale yellow color. Moreover, AmB-PTM-NIO-Gel demonstrated a viscosity of 31870 ± 25 cP and pH of 5.1 ± 0.15. All these parameters were compatible with an ideal topical formulation (Das & Wong, 2020; Andleeb et al., 2021). The drug content of AmB and PTM was found to be 93.45 ± 1.39% and 89.86 ± 1.28%. Similarly, blank gel was clear, uniform, colorless had a pH of 5.2 ± 0.13 and viscosity of 31200 ± 32 cP as reported in Table S1. Additionally, effect of the shear rate on viscosity was also checked by Brookfield viscometer (DV3T, MA, USA) using a CPA 52 Z spindle at room temperature. Obtained results showed that increased shear rate caused shear thinning effect and pseudo plastic flow of the gel leading to decreased viscosity of the AmB-PTM-NIO-Gel (Figure 4B).

### Gel spreadability

The spreadability of both blank and AmB-PTM-NIO-Gel was measured. All the readings were taken in triplicate and results were noted as shown in Table S2. The percentage spreadability of blank and AmB-PTM-NIO-Gel was measured as 313.33 ± 18.92% and 280 ± 26.46%, respectively.

### In vitro release study and kinetic models

**Figure 5.**
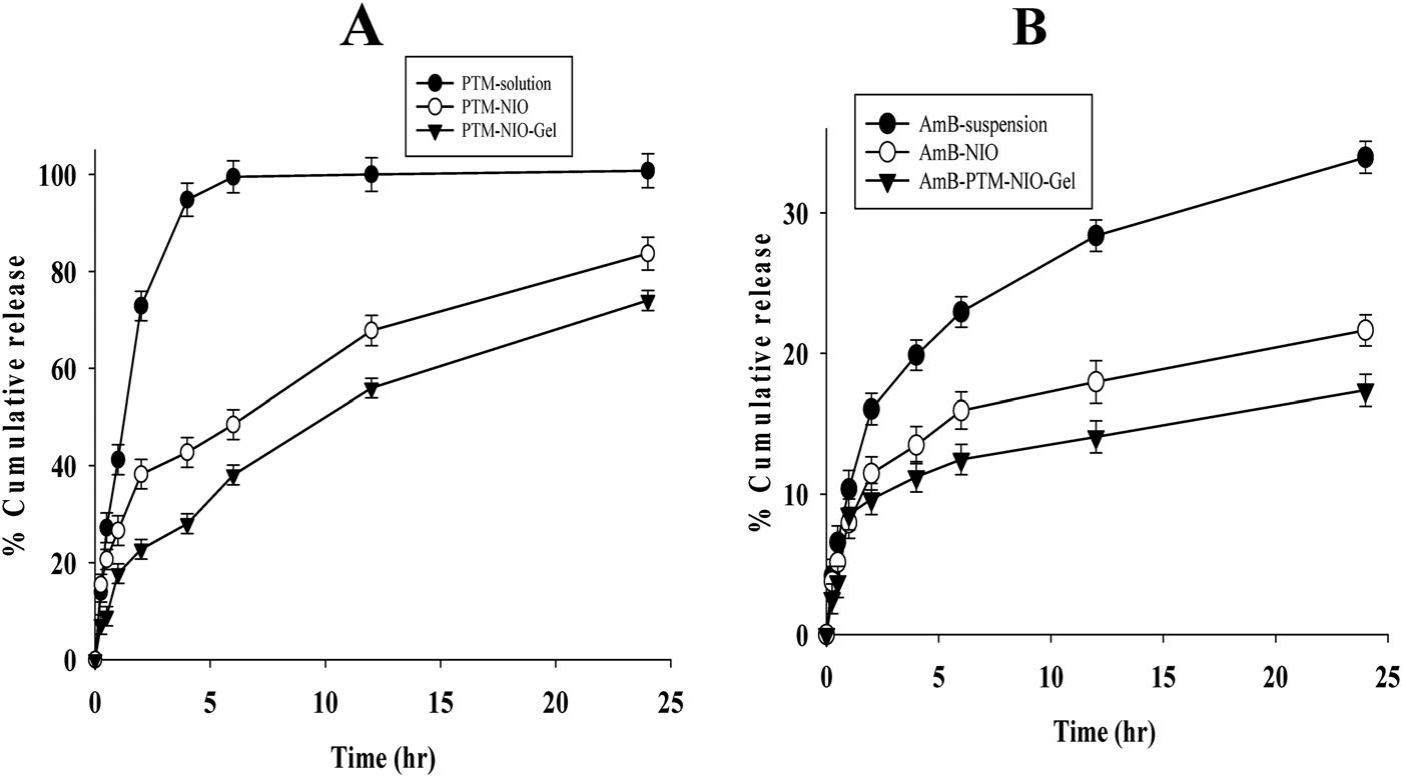
In Vitro release analysis of PTM-NIO, PTM solution and PTM-NIO-Gel **(I);** AmB-NIO, AmB suspension and AmB-NIO-Gel **(II).** Data was analyzed in triplicate (*n* = 3).

*In vitro* release profiles of AmB and PTM from AmB-PTM-NIO-Gel were determined and compared with AmB-PTM-NIO, AmB-NIO, PTM-NIO and the respective AmB and PTM solutions. Analysis was performed at physiological pH of skin macrophages (pH 5.5) and findings of *in vitro* release study are reported in Figure 5 and Table 3. Figure 5(A) represents the release profiles of PTM from AmB-PTM-NIO-Gel, PTM-NIO and PTM solution. Results demonstrated a quick release of PTM from PTM solution as 94% of drug was released from PTM-solution in first 4 hrs, followed by complete release at 5 hrs. PTM-NIO significantly reduces the drug release as only 42% of drug was released in 4 hrs followed by around 80% drug release in 24 hrs. Similarly, the AmB-PTM-NIO-Gel significantly retarded the drug release as compared to PTM solution and PTM-NIO. Only 25% of the drug was released in 4 hrs trailed by 63% drug release in 24 hrs. These results demonstrated a dual control of drug release when the drug was incorporated in niosomes and gel. This phenomenon may enhance the drug retention at their site of application and may enhance the therapeutic effect of the drug. Figure 5(B) shows the drug **r**elease profiles of AmB from AmB solution, AmB-NIO and AmB-NO-Gel at endosomal pH 5.5. The result depicted about 24% of drug release from AmB suspension in first 4 hrs, followed by around 34% of drug release in 24 hrs. In contrast, AmB-NIO showed 13% of drug release in first 4 hrs trailed by a total 21% drug release in 24 hrs. Moreover, AmB-PTM-NIO-Gel further retarded the drug release as compared to the AmB suspension and AmB-NIO, as only 14% of AmB (around 10% drug) was released in 4 hrs and a total of 17% of drug released after 24 hrs. These results exhibited a meaningfully controlled drug release by the gel system as compared to niosomes and drug suspension.

**Table 3.** Kinetic Models of Drugs Release from AmB-NIO, PTM-NIO and AmB-PTM-NIO-Gel.

Models	Model R² values			
	Amphotericin B (AmB)		Pentamidine (PTM)	
	AmB-NIO	AmB-PTM-NIO-Gel	PTM-NIO	AmB-PTM-NIO-Gel
Zero order	0.1528	0.0543	0.3645	0.7352
First order	0.3653	0.3316	0.8955	0.9661
Krosmeyer peppas	0.9788	0.9563	0.9954	0.9946
Higuchi	0.8727	0.8199	0.9407	0.9946
Hixon-Crowell	0.2954	0.2409	0.8708	0.9516

### Kinetic model for drug release

The *in vitro* release profiles of AmB and PTM from AmB-NIO, PTM-NIO and AmB-PTM-NIO-Gel were subjected to different kinetic models (zero order, first order, Korsmeyer-Peppas, Higuchi, Hixon-Crowell) in order to investigate release kinetics. The R^2^ values for various models are shown in the Table 3. By analyzing R^2^ values of all models, Korsmeyer-Peppas was considered as most fitting for release of both drugs and showed highest R^2^ values for AmB and PTM in AmB-NIO, PTM-NIO and AmB-PTM-NIO-Gel. The value of ‘n’ is the key factor of this model which confirms that the release of drugs from nanosystem was via Fickian or non-Fickian diffusion. The value of ‘n’ for AmB and PTM in AmB-NIO, PTM-NIO and AmB-PTM-NIO-Gel was found to be 0.333, 0.315, 0.370 and 0.503, respectively. In Table 4, diffusion coefficient (*n* < 0.5) for AmB and PTM in AmB-NIO, PTM-NIO indicated that the release followed quasi-Fickian diffusion model, however in case of AmB-PTM-NIO-Gel the value of ‘n’ was more than 0.5 which indicated a non-Fickian diffusion release pattern of the gel (Paarakh et al., 2018).

**Table 4.** Values of Korsmeyer-Peppas model for drug release.

Korsmeyer-Peppas	AmB-NIO	AmB-PTM-NIO-Gel	PTM-NIO	AmB-PTM-NIO-Gel
R² values	0.9788	0.9563	0.9954	0.9946
Diffusion Exponent (n)	0.333	0.315	0.370	0.503
Release type	Quasi-Fickian diffusion	Quasi-Fickian diffusion	Quasi-Fickian diffusion	Non-Fickian diffusion

### *Ex vivo permeability of PTM* and *AmB*

**Figure 6.**
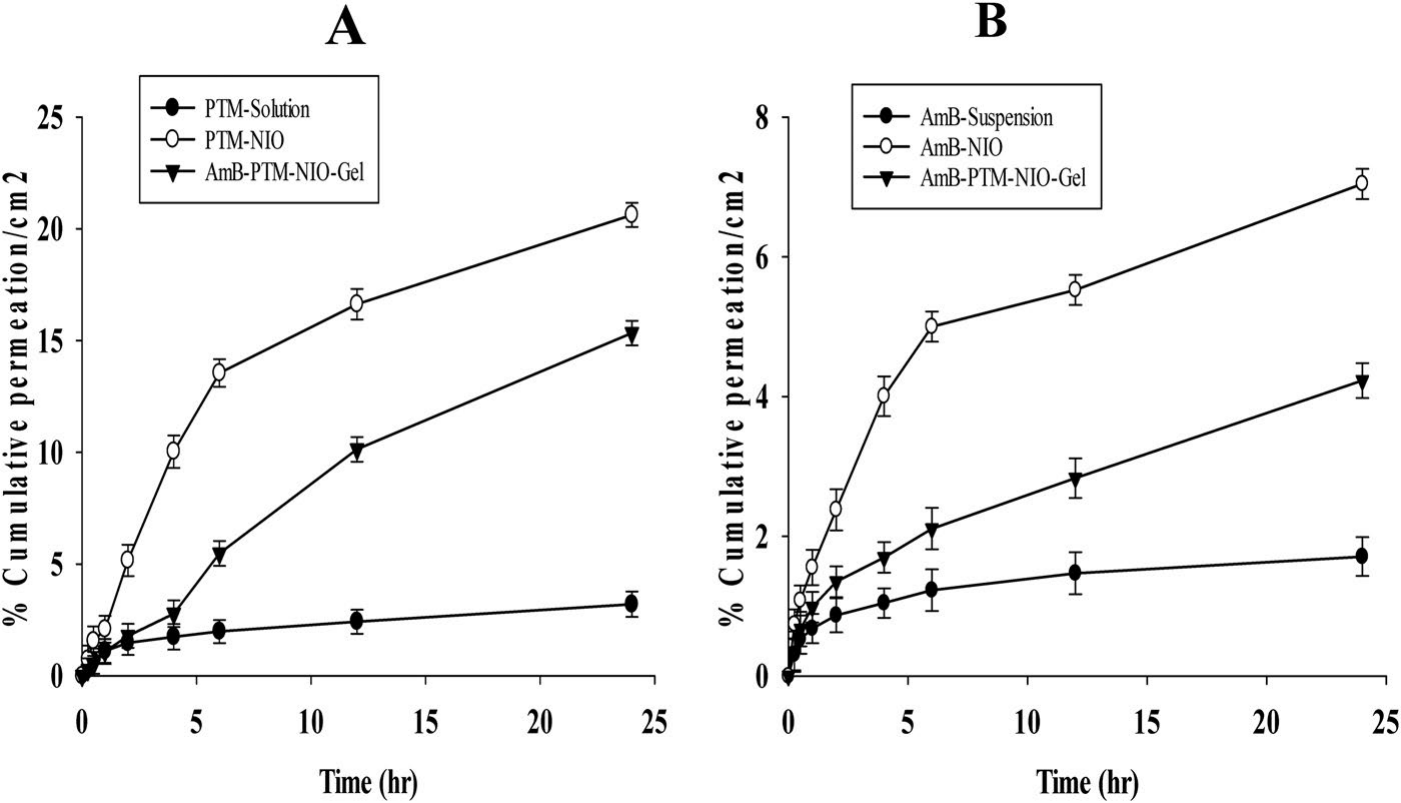
Ex vivo release of PTM-NIO, PTM solution and PTM-NIO-Gel **(I);** AmB-NIO, AmB suspension and AmB-NIO-Gel **(II).** Data was analyzed in triplicate (*n* = 3).

Skin permeation of PTM and AmB from PTM solution, AmB suspension, AmB NIO and AmB-NIO-Gel were performed by using Franz cell diffusion apparatus and results are illustrated in Figure 6(A). A significantly poor permeation of PTM from PTM-solution (3.1%) was observed as compared to its PTM-NIO (22%) (Figure 6A). Whereas, about 15% of PTM was permeated through skin from PTM-NIO-Gel. Similarly, the % cumulative AmB permeated per unit/cm^2^ was measured and reported in Figure 6(B). It was observed, that only 1.7% AmB was permeated through skin from AmB suspension, whereas a significantly enhanced permeation of the drug was observed from AmB-NIO (7%) and AmB-NIO-Gel (4%), respectively, when compared with AmB suspension. These results demonstrated that maximum permeation was obtained from AmB-NIO followed by AmB-NIO-Gel. Moreover, poor permeation was attained from AmB suspension. The stable permeation flux, enhancement ratio and permeability coefficient were also obtained from the *ex vivo* permeability data given in Table S3. The flux and permeability coefficient of AmB-NIO were approximately 5 times higher than the AmB suspension and PTM-NIO flux was almost 6 times higher than PTM-solution. While for the AmB-NIO-Gel and PTM-NIO-Gel flux was approximately 4 times and 5 times higher than the plain drug suspension, respectively. The Kp values of niosomes and NIO-gel were also higher as compared to the pure drugs. In nutshell, these results demonstrated a significantly augmented skin permeation of AmB and PTM when incorporated together in niosomal formulation when comparison with their pure forms.

### Evaluation of skin after treatment

**Figure 7.**
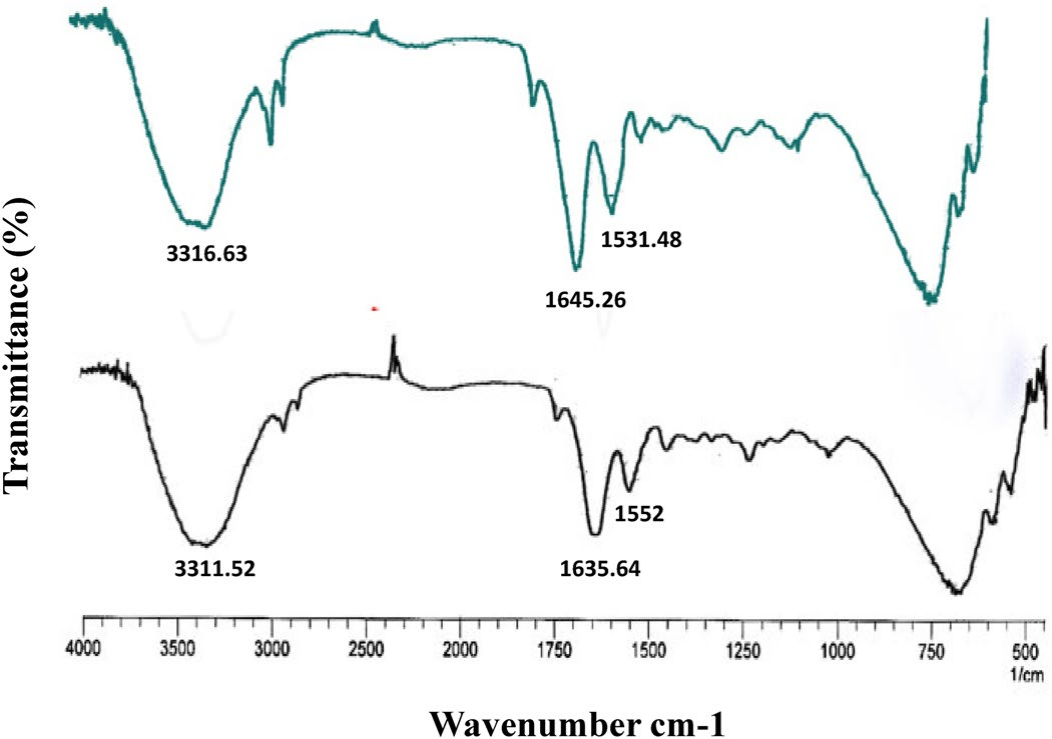
Evaluation of the AmB-PTM-NIO via Fourier-transform infrared spectroscopy analysis and histopathological investigations. **(A)** Fourier-transform infrared spectroscopy of rat skin treated with AmB-PTM-NIO-Gel and untreated epidermis from skin.

It is hypothesized that the application of gel over skin for longer duration may cause damage to the skin layers. Therefore the FTIR analysis of the untreated and AmB-PTM-NIO-Gel treated rat skin was accomplished to evaluate any structural alterations in skin when treated with AmB-PTM-NIO-Gel. The results of skin analysis are reported in Figure 7. As can be seen, the corresponding peaks of hydrocarbon lipid bilayers of skin at 3316.63 and 3316.52 were observed in case of normal (untreated) and AmB-PTM-NIO-Gel treated skin respectively, signifying that no change in the skin structure was observed after the application of AmB-PTM-NIO-Gel.

Moreover, the corresponding amide I (C = O stretching) and amide II (C = N stretching) peaks were observed in 1635 to 1645 region and 1551 to 1532 region for normal and AmB-PTM-NIO-Gel treated skin. Altogether, no significant structural changes were noted in skin the proteins after treating with AmB-PTM-NIO-Gel (Moolakkadath et al., 2018). Additionally, no major change in the skin structure were perceived after the application of AmB-PTM-NIO-Gel.

### In vivo histopathology and skin irritation study

**Figure 8.**
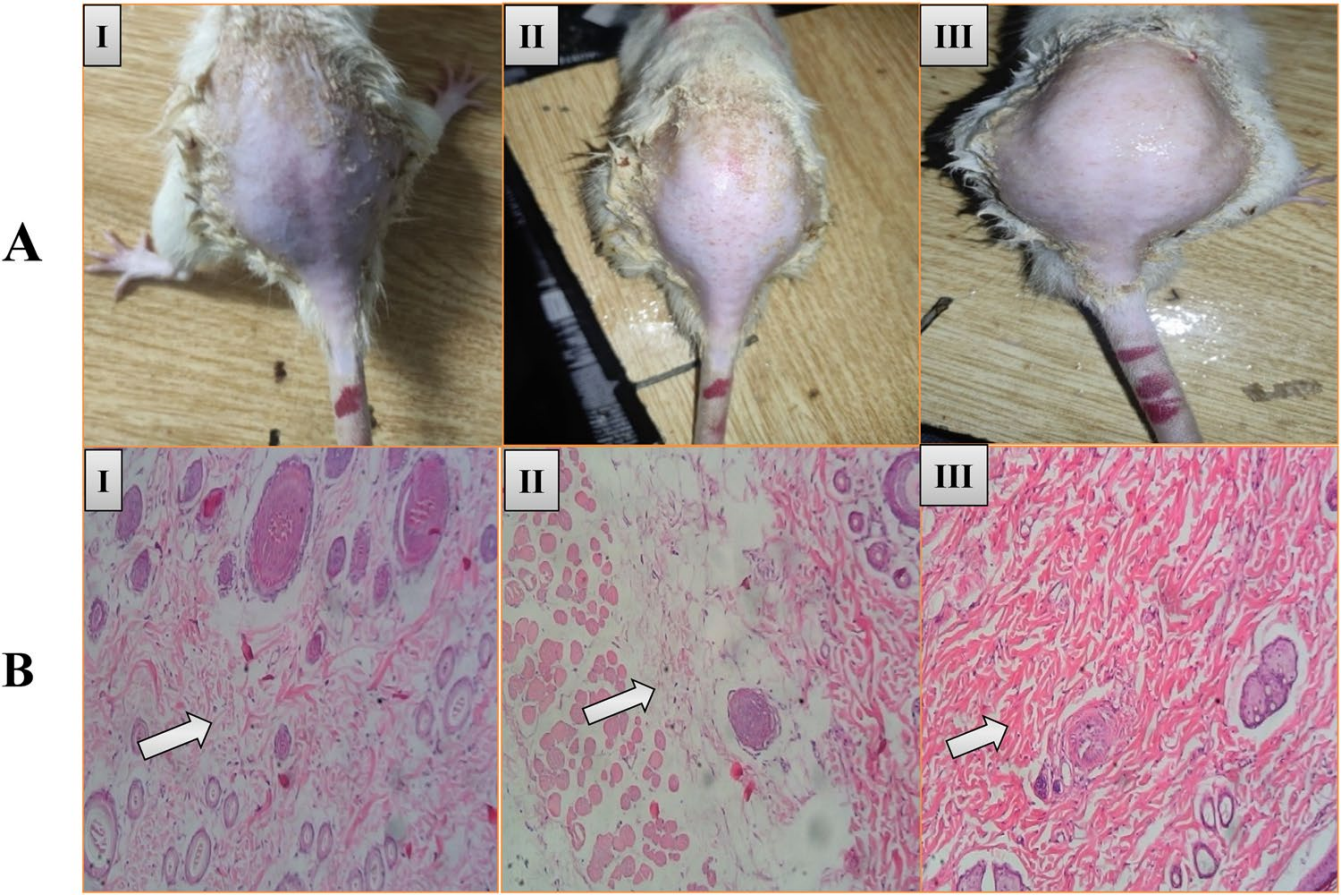
Histopathological results of **(I)** Normal: Skin cells are intact and no sign of infiltration, **(II)** Formalin: Damaged skin cells with infiltration, **(III)** AmB-PTM-NIO-Gel: Skin cells are intact with no sign of infiltration.

To further confirm the results of ex vivo skin safety after treatment with AmB-PTM-NIO-Gel, the histopathology study was performed and compared with normal (untreated), and 0.8% formalin treated skin as shown in (Figure 8A). Fig. 8AI represents the normal untreated rat skin, which indicated no signs of irritation. However, 0.8% formalin treated rat skin have shown major inflammations (Figure 8A II). Similar to normal rat skin, the AmB-PTM-NIO-Gel applied skin didn’t show any major signs of irritation (Figure 8A III). Moreover, histopathology of all the skin samples were performed to find out the actual inflammation on various skin layers (Figure 8B). It was observed that unlike the normal (untreated) rats’ skin (Figure 8BI), the 0.8% formalin treated skin showed major signs of infiltration and damage to the epidermal layer (Figure 8BII). Moreover, the collagen fibers and dermal cells showed severe inflammation and infiltration after 0.8% formalin treatment. Contrary to that, AmB-PTM-NIO-Gel treatment didn’t cause any damage to the rat skin and the dermis and epidermis skin cells remain intact without any visible infiltration and collagen fibers damage (Figure 8BIII). This study demonstrated the safety of the prepared formulation after topical administration.

### In vitro leishmanicidal assay for promastigote

**Figure 9.**
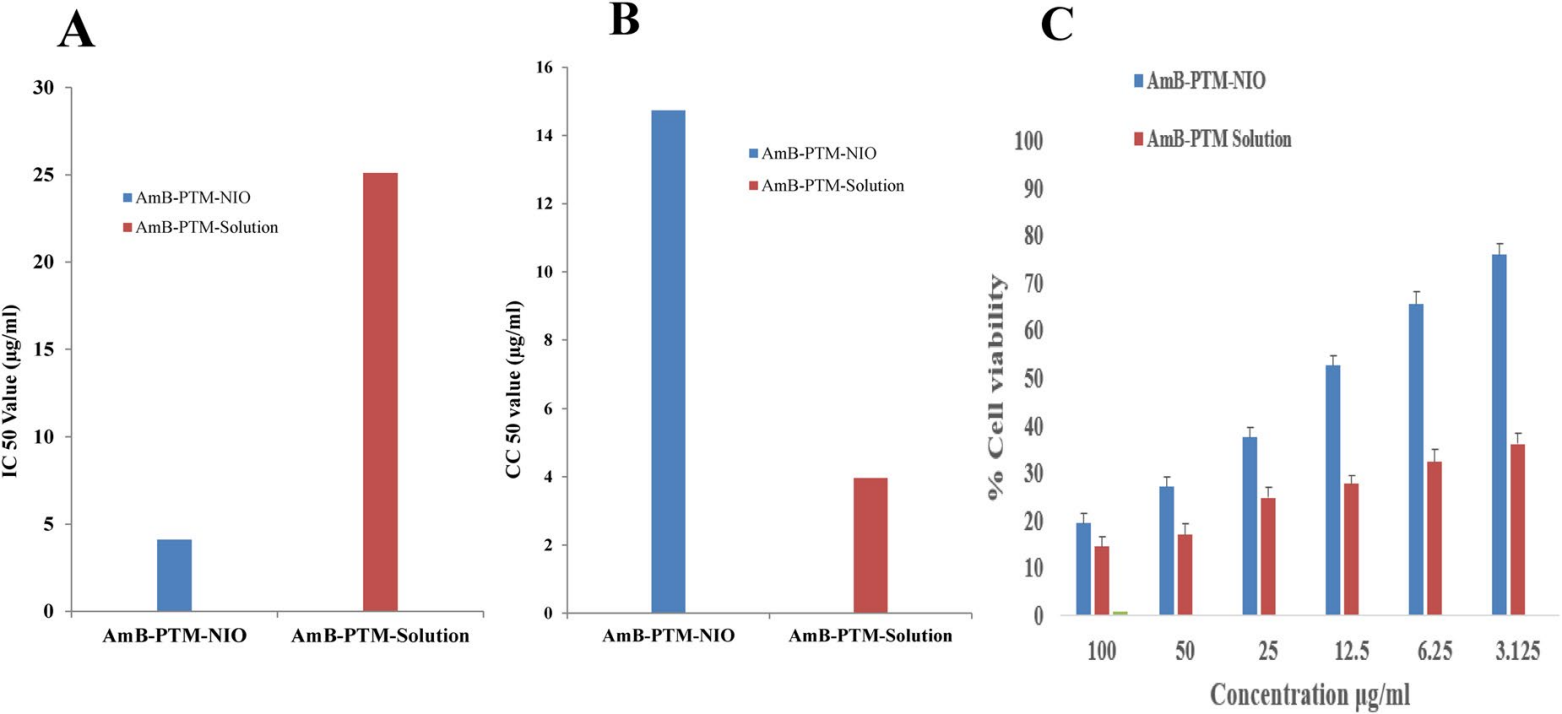
**(A)** Percentage viability at different concentration **(B)** IC_50_ Value of AmB-PTM-NIO and AmB-PTM-Solution **(C)** CC_50_ Value of AmB-PTM-NIO and AmB-PTM-Solution.

The *in vitro* antileishmanial assay of AmB-PTM solution and AmB-PTM-NIO was performed on promastigote and their IC50 values are reported in Figure 9 and Table S4. The IC50 values of AmB-PTM-NIO and AmB-PTM solution were respectively observed as 4.102 ± 0.26 (µg/ml) and 25.10 ± 1.21 (µg/ml), demonstrating a significant difference in their IC50 value. Moreover, a significant difference was noted between the percentage inhibition of AmB-PTM-NIO and AmB-PTM-solution as depicted in (Figure 9A). Similarly, a significantly higher CC50 value of AmB-PTM-NIO (14.73 ± 1.49 µg/ml) was observed as compared with AmB-PTM-solution (3.96 ± 0.65 µg/ml) as indicated in (Figure 9B). These results demonstrated a significantly enhanced antileishmanial potential of the niosomal formulation when compared with the solution (Table S5). Macrophage uptake of the AmB and PTM was quantified by incubating the AmB-PTM-NIO and AmB-PTM-solution in previously harvested macrophages.

Results showed that both AmB and PTM uptake was meaningfully enhanced from the AmB-PTM-NIO as compared to the AmB-PTM solution. Briefly, 23.83 ± 1.25 µg/ml and 68.91 ± 3.19 µg/ml AmB and PTM were uptake by the macrophages respectively from AmB-PTM-NIO. However, only 5.266 ± 0.80 µg/ml and 42.82 ± 2.14 µg/ml AmB and PTM were up taken from the AmB-PTM solution, respectively by the macrophage cells. The results indicated that niosomes have high level of internalization of the drugs as compared to solution Table S5.

### Cell viability and cell uptake study

Percentage cell viability study was also performed by collecting resident macrophages from the rats. It was observed that percentage cell viability in case of AmB-PTM-NIO was more as compared to AmB-PTM solution at every point, such as at a concentration of 3.125 µg/ml viability of cells were 78% in case of AmB-PTM-NIO and about 39% of cells were vial in case of AmB-PTM solution (Figure 9C). GraphPad Prism® was used to calculate the CC_50_ values of AmB-PTM-NIO and AmB-PTM solution. The result showed that CC_50_ value of AmB-PTM-NIO and AmB-PTM solution was 14.73 ± 1.49 and 3.96 ± 0.65 respectively (Table S4) which indicates that AmB-PTM-NIO is safer to use as compared to AmB-PTM solution.

Quantitative macrophage uptake study was performed to determine the extent of engulfment of niosomes by the macrophages. The quantitative uptake of AmB and PTM was quantified by incubating the AmB-PTM-NIO and AmB-PTM solution with previously harvested macrophage cells. In AmB-PTM solution, only 5.266 ± 0.80 and 42.82 ± 2.14 of AmB and PTM were found in the lysed cells respectively. However, in the case of AmB-PTM-NIO, this quantity was enhanced upto 23.83 ± 1.25 and 68.91 ± 3.19 for AmB and PTM respectively (Table S5). This study confirmed the significantly increased uptake of AmB and PTM by macrophages when loaded into niosomes as compared to the AmB-PTM solution. This could be attributed to the within range (< 200 nm) size of the AmB-PTM-NIO, which facilitate the macrophage uptake. Moreover, the macrophage enriched internalization of AmB-PTM-NIO also enhance the antileishmanial potential of both AmB and PTM in leishmanial parasites.

### Stability studies

Stability study of the AmB-PTM-NIO was performed for a period of three months at 4 °C and 30 °C and its findings are reported in Table 5. AmB-PTM-NIO were assessed for PS, PDI and ZP. Stability data showed that a non-significant increase was observed in PS from 228.12 ± 1.39 nm to 235.2 ± 1.14 nm (at 4 °C) and 239.34 ± 5.68 (at 30 °C) respectively. Similarly, in case of PDI and ZP there was negligible variation during storage conditions. The initial PDI and ZP values of optimized formulation were 0.195 ± 0.05 and −36.32 ± 1.94 mV and after duration of three month, it was found to be 0.232 ± 0.02 and −34.28 ± 1.37 correspondingly at 4 °C and 0.258 ± 0.05 and −32.59 ± 3.22 respectively at 30 °C.

## Discussion

CL is an infectious dermal disease characterized by myalgia, abdominal pain, pancreatitis, arthralgia, renal tubular acidosis, nephrotoxicity and cardiovascular effects. There are several alternatives for treatment of CL; nevertheless, many medicines are discontinued due to the emergence of leishmania resistance, gastrointestinal cramps, renal impairment, cardiac arrhythmias, liver disorders, convulsions, peripheral neuropathy and thrombophlebitis (Rabia et al., 2020). Systemic delivery of anti-leishmanial drugs is associated with severe side effects therefore, development of smart nano delivery system is required which is effective and safe for the treatment of CL (Jamshaid et al., 2023). Treatment strategies of CL with monotherapies is associated with many challenges such as drug resistance, cytotoxicity and sensitivity thus combination of suitable candidates is considered as most effective approach (Salim et al., 2020). Thus, co-administration of the antileishmanial drugs is highly recommended for the treatment of CL (Khan et al., 2023). In the past, numerous studies have discussed combination therapy for the topical treatment of numerous diseases such as vitiligo, skin cancer and skin inflammation. Combination therapy surpasses monotherapy even at much lower doses, that’s why it is considered as the most effective and advantageous therapy. The main objective of current study was to develop a co-loaded nano vesicle system for the targeting of Leishmania parasites that resides in the deeper layer of skin (Steverding, 2017). For this purpose, AmB and PTM were selected owing to their antileishmanial efficacy. Topical route was selected for the delivery of co-loaded nano vesicle, which is WHO recommended route for the treatment of CL (Salim et al., 2020). SC is the main hindrance in the delivery of drug through topical route, because it has tough layer of packed cells that inhibits the entry of foreign particles. Therefore, nanoparticles i.e. niosomes were selected as carrier which can easily enter through SC and deliver drug at targeted site (Vora et al., 1998; Zeb et al., 2019). AmB and PTM were selected as best candidate for the treatment of CL. AmB kills parasites by creating pores in the cell membrane and considered as best candidate for the treatment of CL. Inhibition of topoisomerase is the most accepted mechanism of PTM. In accordance with WHO recommendations, a drug combination was loaded into nanoparticles, which not only lowers the probability of resistance but also shortens the course of treatment (Jamshaid & Khan, 2021).

Niosomes, usually composed of lipids, nonionic surfactants and drug which are biodegradable and safe for use in biomedicine (Wagh & Deshmukh, 2010; Bartelds et al., 2018). Different methods are used for the preparation of niosomes including, ether injection method (Chen et al., 2019), reverse phase evaporation method (Thabet et al., 2022), sonication (Ge et al., 2019) method and thin film hydration method (Bhardwaj et al., 2020). Among all methods, thin film hydration is an efficient and easy approach which is frequently used to produce niosomes. They are highly appraised topically used drug delivery systems, as they improve drug residence time at SC and epidermal layers of skin and minimizes the systemic availability of drug, leading to improved biopharmaceutical performance of the incorporated drugs (Shirsand et al., 2012). Membrane rigidity in niosomes is achieved by use of appropriate amount of cholesterol. Niosomes are formed by assembling of nonionic surfactants. A number of advantages of niosomes are reported over liposomes including better stability and longer shelf life. Moreover, they are less toxic and their surface can be modified easily via the functional groups (Chen et al., 2019). Numerous studies have found that the presence of hydrophilic, amphiphilic, and lipophilic moieties in the structure of niosomes makes them one of the best drug delivery systems among all carriers because they can accommodate drug molecules with a wide range of solubility (Khatoon et al., 2017; Bahraminejad et al., 2022).

Optimization of AmB-PTM-NIO was done by using Box-Behnken design of Design Expert^®^ software. Cholesterol, Span^®^ 80 and Span^®^ 60 were selected as independent variables while PS, PDI and ZP were kept as dependent variables. PS and PDI plays key role in efficient permeation of nano carriers across the SC. Nano carriers with PS less than 300 nm can easily deliver drugs in the deeper layers of skin. It was found that by increasing the concentration of cholesterol the PS reduces significantly which could be justified by the fact that increase in concentration of cholesterol leads to increase in the bilayer hydrophobicity which in turn causes decrease in surface free energy resulting in reduction of particle size (Nowroozi et al., 2018). It was observed that by increasing the concentration of span^®^ 60 and span^®^ 80 PS reduces, as combination of span^®^ 60 and span^®^ 80 decreases the particle surface free energy leading to reduction in PS (Gugleva et al., 2019). Cholesterol showed a significant negative effect on PDI and same result were reported by (Nayak et al., 2020) and (Talebi et al., 2021) in their study. With an increase in cholesterol concentration, PDI reduced and niosomal population leads to more homogeneity. Cholesterol also prevents the niosomal aggregation by addition of molecules which stabilizes the system and prevents formation of aggregates by repulsive forces or electrostatic forces. Effect of span^®^ 60 and span^®^ 80 on PDI was similar to cholesterol and PDI near to zero leads to more segregation of particles resulting more stable nano system. It was observed that increase in concentration of cholesterol causes reduction in ZP. Similar results were reported by (Akbari et al., 2020; Zhang et al., 2020) in their study. Moreover, nonionic surfactant didn’t show significant effect on the ZP because of their neutral nature.

TEM analysis showed that AmB-PTM-NIO were spherical in shape; single layered and well segregated indicating low PDI with uniform size (ud Din et al., 2015). TEM analysis also verified that the optimized formulation was monodispersed as validated by DLS analysis by utilizing Zetasizer (Jamshaid et al., 2022; Maqsood et al., 2022). Additionally, no sign of particle aggregation was observed, which points low PDI that indicates stability (Khan et al., 2023).

Bio-adhesive gels offer easy application, and localization as well as good accessibility and self-dosage ease (Sabir et al., 2019; Ali et al., 2022). Chitosan is part of the pseudo natural cationic polymer group and is derivative of chitin. It can be conjugated to various functional groups via complex formations such as with alkyls, metals, sulfates, carboxyl, cyclodextrins, phosphoryl – among others. It is freely soluble in water, particularly at acidic pH and is therefore utilized to make films and gels (Valenta 2005). In addition, chitosan also possesses a mucoadhesive property and enhance the release rate of the loaded drugs (Berger et al., 2004). Chitosan loaded AmB-PTM-NIO gel was prepared and investigated for physicochemical characteristics. The gel was found to be yellowish, translucent, with suitable gelling properties including shear thinning effect which is well desired for topical application (Mushtaq et al., 2022; Zahid et al., 2022). Moreover, the gel appeared to be non-gritty because of the homogeneous dispersion of nanoscale particles. AMB-PTM-NIO-Gel was prepared because of its excellent skin retention properties, which can facilitate the prolonged release of drugs at the lesion site (Khan et al., 2023). pH of gel was found to be 5.1 ± 0.15 which is in range of normal skin pH i.e. 4–6 (Alves et al., 2005). Viscosity of gel decreases due to increase in share rate resulting share thinning effect & pseudo plastic flow of gel, which is suitable for topical administration (Chawla & Saraf 2012). Spreadability is a crucial factor which ensures that gel is administered consistently to the applied area (Kumbhar et al., 2013). AmB-PTM-NIO loaded gel showed optimum spreadability which is suitable for the topical application.

The rate of drug release from nanocarriers is crucial factor for an optimal system, as it represents the concentration and duration of drug available at the targeted site (Bae & Park 2011). In-vitro release study was carried out on physiological pH of skin macrophages (5.5) and blood (7.4) to analyze the release pattern of both drugs AmB and PTM. The obtained results showed that almost 94% of PTM from PTM-solution and 24% of AmB from AmB suspension were released in first 4 hrs while in case of AmB-PTM-NIO and AmB-PTM-NIO loaded gel the release of both drugs was sustained upto 24 hrs. The in-vitro release profile of AmB-PTM-NIO Gel showed a prolonged release of the drug, which avoid frequent application of gel. PTM showed initial burst release followed by sustained release behavior from AmB-PTM-NIO Gel as compared to AmB-PTM-NIO and PTM solution (ud Din et al., 2015; 2017). The initial burst release of the PTM could be due to its desorption on the surface of vesicles while afterwards sustain release pattern is because of diffusion of drug through bilayer (Heidari et al., 2020). AmB-PTM-NIO Gel showed sustain release pattern because the drugs pass through two barriers, first through the niosomes and then through the chitosan gel (Salim et al., 2020). The sustained release pattern from AmB-PTM-NIO as compared to AmB-PTM-NIO-Gel can be attributed to the fact that gels have ability to form a mesh-like matrix system from which the drugs diffuse out, thus drugs have an additional barrier (Zheng et al., 2016). To analyze the release profile of AmB and PTM from niosomes and niosomal gel different kinetic models (First order, zero order, Korsmeyer-Peppas, Hixon-Crowell, Higuchi) were applied by utilizing DD-solver (Microsoft Excel Add-in). By analyzing R^2^ values of all models, Korsmeyer-Peppas model was considered as most suitable model for the AmB and PTM in vitro release from AmB-NIO, PTM-NIO and AmB-PTM-NIO-Gel. The kinetic model data also depicted a prolonged release of both the drugs.

The ex-vivo permeation study provides essential information regarding the behavior of AmB-PTM-NIO in an in-vivo setting by quantifying the amount of drug accessible to the body. Results of skin permeation study depicted that the permeation of drugs from the solution and suspension was very low as compared to the AmB-PTM-NIO formulation. This could be because the niosomes can cross the skin main barrier i.e. SC very easily as compared to free drug (Junyaprasert et al., 2012). The higher values of flux, Kp and enhancement ratio of niosomal formulation as compared to drug solution showed that there is significant difference between the permeation of drugs in case of free drug solutions and niosomal formulation. The increase in both drugs flux in case of nanoparticles could be attributed to nano size of particles, which has been reported to enhance drug penetration (Try et al., 2016). The considerable increase in permeation flux and ER value may be attributable to improved capacity of nanoparticles to penetrate epidermal barriers when compared to pure drugs (Choudhury et al., 2019).

FTIR analysis were done to find out the integrity of SC as it can be affected by nanocarriers (Zeb et al., 2016). The proteins and lipids present in the SC show molecular vibrations at various wavenumbers. Some major peaks are present at 3000 cm^−1^, 2850 cm^−1^, and 2920 cm^−1^ which represents the stretching of alky groups, symmetric stretching of CH_2_, and asymmetric stretching of CH_2_ in lipid hydrocarbon chain respectively. Moreover, peaks at 1550 cm^−1^ and 1650 cm^−1^ corresponds stretching of amide bonds of proteins of SC (Obata et al., 2010). Any alterations in structure of SC resulting in shifting of CH_2_ stretching to higher wave numbers (Shaji & Lal 2014).

Liposomes and various other polymeric nanoparticles alter the fluidity and stability of skin lipids especially in SC, as they pass through skin. In order to determine whether AmB-PTM-NIO had any impact on the lipids in the skin layers, skin FTIR analysis was carried out. FTIR analysis of skin depicted no major change in the transmittance of normal and AmB-PTM-NIO-Gel, demonstrating that AmB-PTM-NIO-Gel didn’t disrupt the skin layers and its integrity was maintained (Rabia et al., 2020).

Any dosage form designed for topical application must be nontoxic and nonirritating. In order to verify the safety profile of AmB-PTM-NIO, skin irritation study was performed, and results were compared with standard skin irritant (0.8% formalin). This analysis demonstrates that AmB-PTM-NIO-Gel provides a better safety profile. Results of skin irritation study were further confirmed by histopathology analysis and showed that epidermal layers of skin treated with AmB-PTM-NIO-Gel remained intact with no sign of infiltration and loose collagen fibers (Bibi et al., 2022). Thus, the findings of skin irritation study confirmed that AmB-PTM-NIO-Gel was appropriate for topical administration and exhibited no sign of skin irritation.

In-vitro anti-leishmanial assay showed that IC50 value of AmB-PTM-NIO lowered to great extent as compared to the plain drugs solution and found more effective. The result proved that the combination of suitable agents has proficient effect (Salim et al., 2020). Cell cytotoxicity and viability study data proved that AmB-PTM-NIO nanoparticles are safer to use in contrast to plain drugs solutions. This is due to the fact that niosomal drug formulations have been shown to be effective with controlled drug delivery systems and they also provide successful drug localization in skin with more efficacy, higher stability and lower adverse effects (Parizi et al., 2019).

One of the main hurdle toward effective therapy is incorporation of drugs inside macrophages where leishmanial parasites reside (Romero & Morilla 2008). Since, the Leishmania parasite resides inside skin macrophages, the effectiveness of an anti-leishmanial formulation mainly depends on how efficiently it targets and accumulates there (Shahnaz et al., 2017). Macrophage uptake study was performed to investigate internalization of AmB-PTM-NIO in deeper layers of skin and release of drugs at targeted site. Macrophage internalization assay results indicated that uptake of AmB-PTM-NIO was superior in contrast to the drug solution or suspension form, thus ensuring effective killing of *Leishmania* parasite at targeted site. Increased internalization of nano vesicles can be due to reduced size of the nano carriers (Mir et al., 2017; ud Din et al., 2017). In addition, enhanced internalization of nano particles improves the efficacy against the leishmanial parasites as well as might act as drugs depot and improves drug availability to leishmanial parasites residing in macrophages (Jamshaid & Khan, 2021). Though since infected macrophages are the preferred site of action for anti-leishmanial drugs, thus due to favorable vesicle characteristics and successful localization of nanocarriers it was anticipated that anti-leishmanial efficacy and potential of AmB and PTM could be substantially enhanced when loaded within NIO (Borborema et al., 2011).

Stability study results revealed that AmB-PTM-NIO formulation was stable at both temperatures and no substantial difference in the PS, ZP and PDI was observed at storage conditions. Besides, no sign of precipitation and particle aggregation was evident throughout the evaluation period.

## Conclusions

The AmB-PTM-NIO were statistically designed, and developed with excellent particle properties including nano sized, mono dispersed and stable vesicles. The optimized AmB-PTM-NIO were loaded into the chitosan-based gel system to form AmB-PTM-NIO-Gel to enhance retention time. The niosomal gel had suitable homogeneity, spread ability and desired viscosity for topical application. Additionally, AmB-PTM-NIO and AmB-PTM-NIO-Gel displayed a prolonged release behavior of the incorporated drugs in in vitro release experiments. Moreover, the enhanced cutaneous flux demonstrated by AmB-PTM-NIO-Gel was beneficial for retaining drugs in deeper skin layers for a longer duration of time. The outcomes of skin permeation study for AmB-PTM-NIO and AmB-PTM-NIO-Gel indicated the higher permeability of niosomes into the skin for topical effect. In addition, in vivo skin irritation and histopathological evaluation of the developed formulation exhibited its safety after skin application. Furthermore, a significantly improved percent inhibition and meaningfully reduced IC_50_ values of the AmB-PTM-NIO showed its augmented antileishmanial potential in *L. tropica* promastigotes. Beside this, a profoundly enhanced cellular uptake of the AmB-PTM-NIO by the targeted macrophage showed a well desired internalization of drugs in the infected cells. The cell viability and toxicity study also indicated the safety of AmB-PTM-NIO. All the findings were strongly in favor of considering AmB-PTM-NIO as a viable anti-leishmanial candidate.

## Supplementary Material

Supplemental Material

## Data Availability

Data will be made available on request.
