# Supplementary material for: Co-delivery of amphotericin B and pentamidine loaded niosomal gel for the treatment of Cutaneous leishmaniasis
Source: Drug Deliv. 2023 Feb 1;30(1):2173335. doi: 10.1080/10717544.2023.2173335 (PMC9897754; doi:10.1080/10717544.2023.2173335)
Supplement: Supplemental Material [file IDRD_A_2173335_SM6443.docx]

**Co delivery of amphotericin B and pentamidine loaded niosomal gel for the treatment of cutaneous leishmaniosis**

Supplementary Document

Figure 1.


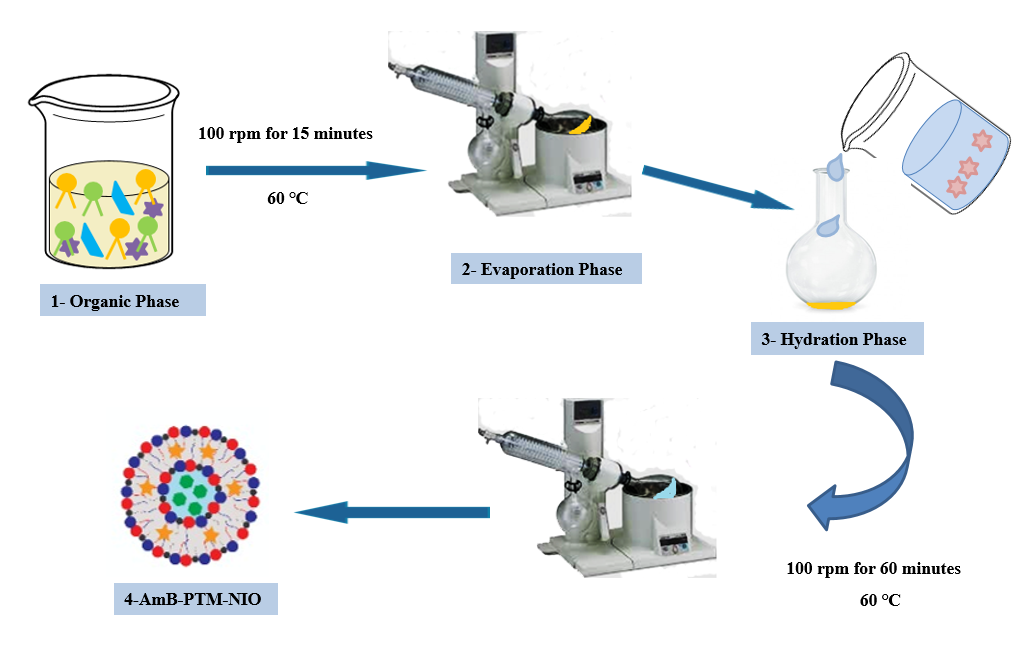


**Table S1: Characterization of AmB-PTM-NIO-Gel and Blank Gel**

| **Parameters** | **AmB-PTM-NIO-Gel** | **Blank Gel** |
| --- | --- | --- |
| pH | 5.1 ± 0.15 | 5.2 ± 0.13 |
| Appearance | Clear opaque | Transparent |
| Homogeneity | Uniform | Uniform |
| Color | Pale yellow | Colorless |
| Viscosity (cP) | 31870 ± 25 | 31200 ± 32 |
| Spreadability (%) | 280 ± 26.46 | 313.33 ± 18.92 |

**Table S2:** Spreadability of blank and AmB-PTM-NIO-Gel

| **Sr. No.** | **Blank gel** | | | **AmB-PTM-NIO-Gel** | | |  |
| --- | --- | --- | --- | --- | --- | --- | --- |
|  | **Initial area (cm)** | **Final area (cm)** | **Spreadability %** | **Initial area (cm)** | **Final area (cm)** | **Spreadability %** | |
| 1 | 2 | 6.7 | 335 | 2 | 6 | 300 | |
| 2 | 2 | 7 | 350 | 2 | 5.8 | 290 | |
| 3 | 2 | 6 | 300 | 2 | 5 | 250 | |
| Mean ± S.D. | | | 313.33 ± 18.92 |  |  | 280 ± 26.46 | |

**Table S3: Skin Permeation Parameters**

| **Formulation** | **Cumulative amount permeated in 24 hrs. (μg/cm^2^ )** | **Jss**  **(μg/cm^2^/h)** | **Kp**  **(cm^2^/h)** | **ER** |
| --- | --- | --- | --- | --- |
| AmB dispersion | 34.18 ± 2.25 | 1.42 ± 0.12 | 0.00071 ± 0.0001 | - |
| PTM solution | 57.60 ± 3.88 | 2.39 ± 0.25 | 0.0013 ± 0.0009 | - |
| AmB-NIO | 140.89 ± 6.89 | 5.87 ± 1.61 | 0.0029 ± 0.002 | 4.12 ± 1.22 |
| PTM-NIO | 309.41 ± 5.37 | 12.89 ± 1.17 | 0.0085 ± 0.001 | 5.37 ± 1.89 |
| AmB-NIO-Gel | 105.77 ± 4.35 | 4.40 ± 0.91 | 0.0017 ± 0.0005 | 3.09 ± 0.94 |
| PTM-NIO-Gel | 276.10 ± 5.12 | 11.50 ± 1.084 | 0.0063 ± 0.0009 | 4.79 ± 1.18 |

Here ±: Standard Deviation (n=3); Jss: Steady State Flux; Kp: Permeability Coefficient; ER: Enhancement Ratio.

**Table S4: IC_50_ & CC50 values of formulation solution and formulation**

| **Assay** | **AmB-PTM-NIO** | **AmB-PTM-Solution** |
| --- | --- | --- |
| **IC_50_ value ± S.D (µg/ml)** | 4.102 ± 0.26 | 25.10 ± 1.21 |
| **CC_50_ value ± S.D (µg/ml)** | 14.73 ± 1.49 | 3.96 ± 0.65 |

Here IC_50_: Minimal Concentration of Drug Required for 50% Inhibition In Vitro; CC_50_: The Extract Concentration that Reduced the Cell Viability by 50% ; ± S.D: Standard Deviation (n=3)

**Table S5: Cell uptake study**

| **Cell uptake**  **± S.D**  **(µg/ml)** | **AmB** | | **PTM** |  |
| --- | --- | --- | --- | --- |
|  | **(AmB-PTM-Solution)** | **(AmB-PTM-NIO)** | **AmB-PTM-Solution** | **AmB-PTM-NIO)** |
|  | 5.266 ± 0.80 | 23.83 ± 1.25 | 42.82 ± 2.14 | 68.91 ± 3.19 |

Here ± S.D: Standard Deviation (n=3)
